# Relaxation Phenomena in Chitosan-Au Nanoparticle Thin Films

**DOI:** 10.3390/polym13193214

**Published:** 2021-09-23

**Authors:** Elodie Strupiechonski, Marisa Moreno-Ríos, Erika O. Ávila-Dávila, Ramón Román-Doval, Evgeny Prokhorov, Yuriy Kovalenko, Diana G. Zárate-Triviño, Dora I. Medina, Gabriel Luna-Barcenas

**Affiliations:** 1 CONACYT—Cinvestav Unidad Queretaro, Queretaro 76230, Mexico; elodie.strup@cinvestav.mx; 2 Department of Mechanical Engineering, Tecnologico Nacional de Mexico, Instituto Tecnologico de Pachuca, Pachuca 42083, Mexico; marisa.mr@pachuca.tecnm.mx (M.M.-R.); erika.ad@pachuca.tecnm.mx (E.O.Á.-D.); 3 Tecnológico Nacional de México, Instituto Tecnológico del Valle de Etla, Abasolo S/N, Barrio del Agua Buena, Santiago Suchilquitongo, Oaxaca 68230, Mexico; rrdoval.11@gmail.com; 4 Cinvestav Unidad Queretaro, Queretaro 76230, Mexico; prokhorov@cinvestav.mx (E.P.); kovalenko.yuriy@gmail.com (Y.K.); 5 Laboratorio de Inmunología y Virología, Universidad Autónoma de Nuevo León, Monterrey 64450, Mexico; 6 Tecnologico de Monterrey, School of Engineering and Sciences, Atizapan de Zaragoza, Estado de Mexico 52926, Mexico

**Keywords:** Au-Chitosan nanocomposite, glass transition, relaxation phenomena, dielectric spectroscopy, dynamic mechanical behavior

## Abstract

Chitosan–gold nanoparticle (CS/AuNP) thin films were synthesized through the chemical reduction of HAuCl_4_ in sodium citrate/chitosan solutions. The dielectric and dynamic mechanical behaviors of CS/AuNP films have been investigated as a function of moisture and HAuCl_4_ content. Two relaxation processes in the nanocomposites have been observed. The α-relaxation process is related to a glass transition in wet CS/AuNP films. However, in dry composites (with 0.2 wt% of moisture content), the glass transition vanished. A second relaxation process was observed from 70 °C to the onset of thermal degradation (160 °C) in wet films and from 33 °C to the onset of degradation in dry films. This relaxation is identified as the σ-relaxation and may be related to the local diffusion process of ions between high potential barriers in disordered systems. The α- and σ-relaxation processes are affected by the HAuCl_4_ content of the solutions from which films were obtained because of the interaction between CS, sodium succinate, and gold nanoparticles. With about 0.6 mM of HAuCl_4_, the conductivity of both wet and dry films sharply increased by six orders, corresponding to the percolation effect, which may be related to the appearance of a conductivity pathway between AuNPs, HAuCl_4_, and NaCl.

## 1. Introduction

Chitosan (CS) is a natural polymer derivate of chitin. Its structure comprises glucosamine and N-acetylglucosamine unit residues, and it exhibits properties such as biocompatibility, biodegradability, and low toxicity [1,2,3,4]. Additionally, CS is soluble in aqueous acidic media and demonstrates a good film-forming ability [5,6]. The proper combination of such properties allows for the formation of CS–metal nanoparticle bionanocomposites. Among CS-based nanocomposites, CS/gold nanoparticles (CS/AuNPs) are attractive biocomposites because of their great potential in biomedicine [5,6,7,8,9] and in different biosensor [10,11,12,13,14,15] for detecting protein [16], fetoprotein [17,18] and DNA glucose [16,19,20,21], etc. Several methods have been employed to fabricate Au nanoparticles in a wide variety of shapes and dimensions [22]. The most common methods, however, are based on the reduction of HAuCl4 with sodium citrate (SC) at high temperatures, as first introduced by Turkevitch [23]. The average particle diameter can be tuned over a wide range (from 10 to 100 nm) by varying the concentration ratio between HAuCl_4_ and SC [24]. Typically, nanoparticles tend to aggregate during their synthesis. The literature has shown that using polymers as protecting agents allows fabricating and dispersing Au nanoparticles and prevents their aggregation. Polymers also offer control over the rate of the reduction process and thus enable the production of nanoparticles of different sizes [25]. The possibility of stabilizing Au nanoparticles using CS has been reported in the literature [22,26,27,28,29,30]. As CS in solution is protonated and positively charged, it can be adsorbed onto the surfaces of Au nanoparticles because of the interaction between the amino groups in CS and Au nanoparticles [2,25]. Moreover, a previous study [5] demonstrated that CS stabilizes AuNPs not only in solutions but also in solid films. Such stabilizing and protecting properties of CS for Au nanoparticles can be used to further construct biosensors. These CS–Au nanoparticle films possess interesting structural, chemical, and optical properties that can be tailored for a wide variety of applications, including biosensors [10,11,12,22,31].

Biosensors are a class of devices that produce measurable responses to changes in physical conditions or chemical concentrations. Generally, depending on the type of signal transducer, biosensors generate optical, piezoelectric, or electrical responses. Electrochemical biosensors, which convert biological binding events into useful electrical signals, have received considerable attention in the past years [11,12]. In many cases, AuNPs embedded in three-dimensional CS matrices served as current conductors [11,12,32]. For later applications, the conductivity mechanism of CS/AuNP nanocomposites needs to be addressed. However, the literature has mostly focused on the fabrication, microstructural characterization, and sensibility of CS/AuNPs biosensors [5,6,7,8,9,10,11,12,13,14,15,16,17,18,19,20,21,22,23,24,25,26,27,28,29,30]. The conductive and relaxation properties of CS/AuNP films have not been reported. In this regard, the spirit of our work is to shed light on the relaxation mechanisms governing the proposed nanocomposites that help trigger the development of biosensors, flexible electronics among others.

This work aims to synthesize CS/AuNP films and investigate the influence of Au nanoparticle concentration on the structure, conductivity, and relaxation properties of composites using dielectric spectroscopy, dynamic mechanical analysis (DMA), X-ray diffraction (XRD), thermogravimetric analysis (TGA), Fourier transform infrared (FTIR) spectroscopy, and high-resolution scanning electron microscopy with energy dispersive analysis (EDS).

## 2. Materials and Methods

### 2.1. Synthesis of Nanocomposite

CS with medium molecular weight (300,000 g/mol) and 85% degree of deacetylation (cat. num. 448877), hydrochloroauric acid (cat. num. 50790), and SC (cat. num. S1804) were obtained from Sigma Aldrich^®^ and used without additional purification.

Eleven materials were synthesized with different weight percentages of AuNPs. Nanocomposites were synthesized by dissolving 2% of CS in a 1% acetic acid solution. Thereafter, a HAuCl_4_ solution was added (between 0.01 and 1 mM) and was reduced using a 1% SC solution. The amount of SC added to the reaction was calculated on the basis of the following reaction [32]:

HAuCl_4_ + 4 Na_3_C_6_H_5_O_7_ → Au^0^ + 4 NaCl + 4 Na_2_H_5_C_5_O_5_ + 4 CO_2_ + H^+^ (1)

In theory, 4 mol of SC are necessary for 1 mol of hydrochloroauric acid. CS is a polysaccharide soluble in aqueous acid media below pH 6 [33,34,35,36,37]. At a low pH, amino groups are protonated and have a positive charge, allowing water-soluble polyelectrolytes to form. Conversely, pH values greater than 6 result in CS precipitation. Thus, the amount of SC added for the synthesis reaction should not increase the pH of the solution to above 6. Additionally, the CH_2_ and OH groups of CS may act as reducing groups in the formation of Au nanoparticles [22,26,27,28,29,30]. Thus, in this work, the maximum stoichiometric ratio used is 1 mol of HAuCl_4_ for 2 mol of SC to achieve pH 4.5. SC concentrations were changed along with changes in the amount of HAuCl_4_ in the solution to achieve the same molar relations between HAuCl_4_ and SC.

The solutions were mixed and heated up to 75 °C under magnetic stirring until they turned red.

CS/AuNP films (with thickness ~30 mm) were prepared using the solvent cast method by pouring the final solution into a plastic Petri dish and allowing the solvent to evaporate for 24 h at 60 °C.

### 2.2. Characterization Studies

#### 2.2.1. Infrared Measurements and Morphology Analysis

Materials were characterized by infrared spectroscopy. FTIR spectra between 4000 and 400 cm^−1^ were obtained using an FTIR spectrophotometer (Perkin Elmer Spectrum 1 model, PerkinElmer, Inc. Waltham, MA, USA). All spectra were recorded at 4-cm^−1^ intervals and 16 cm^−1^ times scanning used transmission technique. The morphology of the CS/AuNP films was analyzed using a JEOM JSM-7401F field emission scanning electron microscope (JEOL Inc., Peabody, MA, USA). EDS was performed to obtain the weight percentage of Au in the films.

Crystal structure analysis was performed using a Rigaku diffractometer ULTIMA IV (Rigaku Corporation, Tokyo, Japan) with CuKα radiation (λ = 1.5406 Å).

The ultraviolet–visible spectrum (UV–Vis spectrometer Agilent 8453, Agilent Technologies, Santa Clara, CA, USA) was used to determine the sizes of the Au nanoparticles by detecting the maximum absorption band in the visible region.

#### 2.2.2. Thermal Measurements

The amount of free water was determined using TGA (Metler Toledo 851e model, Mettler Toledo, Columbus, OH, USA). Measurements were made using a dry airflow from 25 °C to 300 °C with a 10 °C/min rate. DMA was conducted using a TA Instruments Dynamical Mechanical Analyzer model RSA III (Mettler Toledo, Columbus, OH, USA) in dry airflow. The heating rate was 5 °C/min, the frequency was 0.1 Hz, and the initial strain was 0.1%.

#### 2.2.3. Conductivity Measurements

The temperature dependencies of DC conductivity can be explained depending on the underlying mechanism responsible for the thermal behavior. In this work, three main behaviors are observed and discussed in the “Results” section:

- A non-linear behavior, well described by the Vogel–Fulcher–Tammann (VFT) relationship:
  (2) $$\sigma =\sigma_{0}\exp(-\frac{DT_{0}}{T-T_{0}})$$
  where *σ*_0_ is the pre-exponential factor, *D* is a material constant, and *T*_0_ is the so-called Vogel temperature.
- An Arrhenius-type linear dependance:
  (3) $$\sigma =\sigma_{0}\exp(-\frac{E_{a\sigma }}{RT})$$
  where $E_{a\sigma }$ is the activation energy.
- A negative slope for temperatures greater than a limit value corresponding to the beginning of degradation [38,39].

A nonlinear dependence before the percolation threshold has been observed in many polymers and can be explained using the variable range hopping (VRH) model proposed by Mott [40,41,42] well described by Equation (5). According to this model, the dependence of DC conductivity on temperature *T* can be written as:

(4) $$\sigma_{dc}(T)=\sigma_{0}\exp\left[-{\left(\frac{T_{0}}{T}\right)}^{\gamma }\right]$$

where *σ*_0_ can be considered as the limiting value of conductivity at infinite temperature and *σ*_0_ ~ 1/*T*^1/2^ [43,44], *T*_0_ depends on the localization and density of the states, and the exponent γ is related to the dimensionality d of the transport process via the equation γ = 1/(1 + *d*), where *d* = 1, 2, 3.

#### 2.2.4. Dielectric Measurements

Dielectric measurements in the frequency range from 40 Hz to 110 MHz were performed using the Agilent Precision Impedance Analyzer 4249A. The amplitude of the measured signal was 100 mV. Temperatures were measured in the cell from 20 °C to 200 °C using a temperature controller, which was programmed to produce a constant heating rate of 3 °C/min between certain measured temperatures. Each sample was kept for 3 min at each temperature to ensure thermal equilibrium. Additional measurements were conducted in a vacuum cell to remove moisture from the films. As-prepared samples were annealed into the vacuum cell before measurements at 120 °C for 1 h, followed by cooling at room temperature in the vacuum. Additionally, a Peltier heating element was used to perform measurements from 0 °C to 100 °C.

Dielectric relaxation measurements can provide additional information on temperature relaxation processes in nanocomposites. Generally, in composites with conductive inclusions, ionic current and interfacial polarization could often mask the real dielectric relaxation processes in the low-frequency range. Therefore, for analyzing the dielectric process in detail, the complex permittivity ε* was converted to the complex electric modulus M* using the following equation:

(5) $$M^{*}=\frac{1}{\varepsilon^{*}}=M^{\prime }+iM^{\prime \prime }=\frac{\varepsilon^{\prime }}{\varepsilon^{\prime 2}+\varepsilon^{\prime \prime 2}}+i\frac{\varepsilon^{\prime \prime }}{\varepsilon^{\prime 2}+\varepsilon^{\prime \prime 2}}$$

where *M*′ is the real part and *M*″ the imaginary part of the electric modulus and *ε*′ is the real part and *ε*″ the imaginary part of permittivity.

Interpreting the experimental data in this form is commonly employed to obtain information on the relaxation processes in ionic conductive materials and polymer–conductivity nanoparticle composites. In this representation, interfacial polarization and electrode contributions are essentially suppressed [45,46]. The corresponding relaxation time can be calculated using the relation τ = 1/(2*πfp*), where *fp* is the peak frequency in the dependence of *M*″ on frequency [35]. The position of the peak in the imaginary part of *M*″ depends on the temperature.

#### 2.2.5. X-ray Diffraction

The XRD test consisted of a material being hit by a beam, and then the intensity and scattering angle is recorded by a detector. XRD were performed to identify in function of temperature the crystalline phase. Water content was also determined from the crystalline morphology. XRD measurements allowed to find out the concentration of HAuCl4 and temperature change of the nanocomposites crystalline structure due the change water content.

## 3. Results

### 3.1. Infrared Spectroscopy

**Figure 1:**
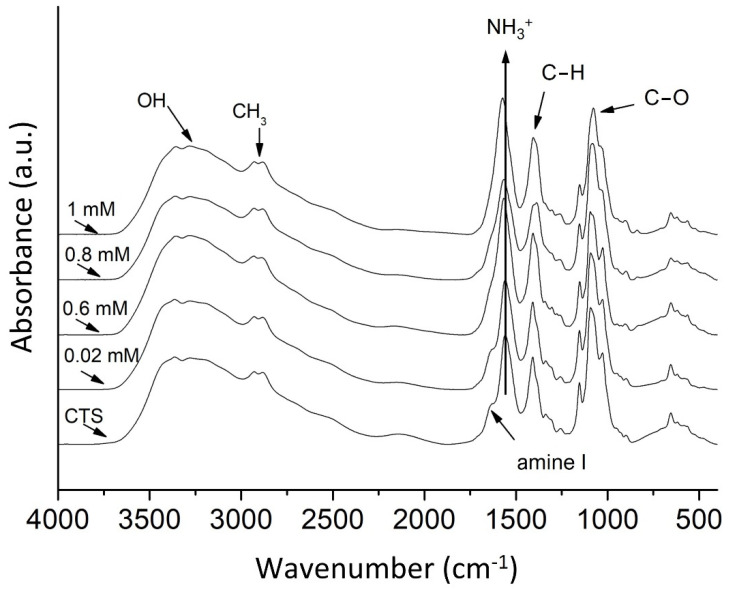
FTIR spectra of the CS/AuNPs films obtained from solution with HAuCl_4_ concentration indicated on graphs.

Figure 1 shows the IR spectra of the CS–Au nanocomposite with different concentrations of AuNPs. The spectra of the materials show a few contributions. The band at 3300 cm^−1^ is produced by symmetric OH groups present in the structure. The band at 2880 cm^−1^ is associated with a symmetric methyl group. The band detected at 1640 cm^−1^ is attributed to a C=O antisymmetric amide I. The band at 1550 cm^−1^ shows a symmetric and antisymmetric deformation of NH_3_^+^. The band at 1410 cm^−1^ is produced by a C–N stretch. The band observed at 1025 cm^−1^ is ascribed to a C–O stretch [7,8,9].

The FTIR spectra of the nanocomposites show fine but significant changes compared with those of the neat CS. The characteristic bands of CS for all materials, though, at 1648 cm^−1^ shifted and decreased the band intensity, which can be ascribed to an amide I. For samples with the highest percentage of nanoparticles, this band almost disappears. This behavior suggests an interaction of the amide group of CS with the products of synthesis such as sodium succinate. The band at 1550 cm^−1^ slipped to higher wavenumbers, corresponding to the deformation of the NH_3_^+^ group. The shift was higher for samples with higher concentrationns, indicating an interaction of the amine group with metal particles [36].

### 3.2. Scanning Electron Microscope Measurements

**Figure 2:**
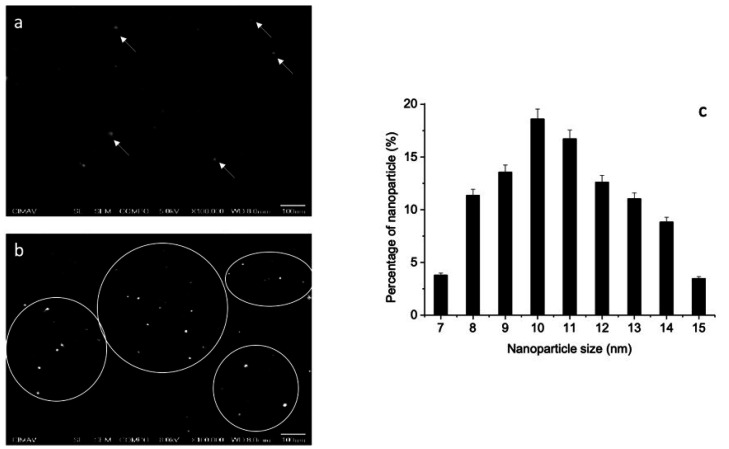
SEM micrographs of films obtained from solution with 0.06 (**a**) and 1 mM (**b**) of HAuCl_4._ white points-gold nanoparticles. (**c**) The particle size distribution histogram obtained from SEM micrographies.

The SEM image (Figure 2) shows that the AuNPs are homogeneously embedded in the CS matrix. The numbers of AuNPs increased with an increase in the HAuCl_4_ concentration. However, according to our micrograph (Figure 2b), no physical connection between Au nanoparticles is observed even at high the HAuCl_4_ concentration. This observation is important for explication of percolation properties of nanocomposites.

**Figure 3:**
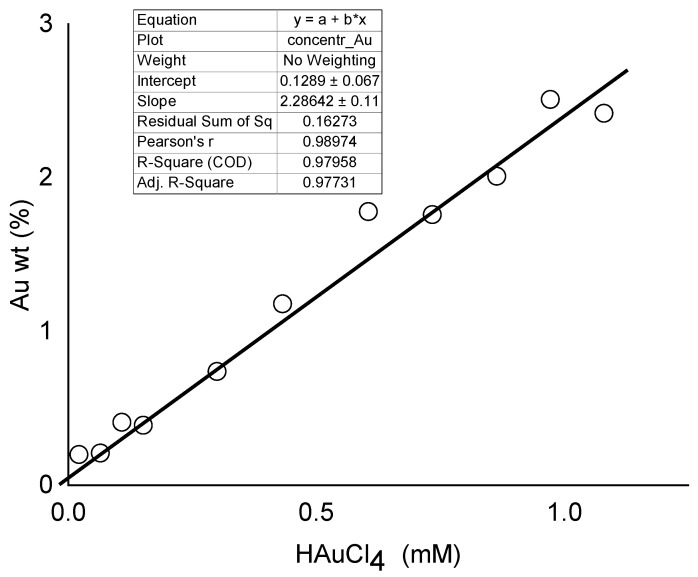
Dependence of gold wt% in the films on the HAuCl_4_ concentration in the solution.

The dimensions of the nanoparticles observed in the SEM micrographs were obtained using the AutoCAD 2007 software. The particle distribution histogram is shown in Figure 3. This histogram was obtained from three micrographs for every investigated film, which have been prepared from solutions with different HAuCl_4_ concentrations. Most of the nanoparticles had sizes between 9 and 12 nm (about 60%, Figure 2). The UV–Vis spectrum demonstrated maximum absorption bands between 530 and 535 nm for all investigated films (not shown). This wavelength range corresponds to an approximate particle size [47,48] obtained from SEM micrography and confirms that particle size did not depend on the HAuCl_4_ and SC concentrations.

As mentioned above, the investigated films have been prepared from solutions with different HAuCl_4_ millimolar (mM) concentrations. From the EDS analysis, the concentrations of Au in the films were obtained. Figure 3 shows the linear dependence of Au (with R^^2^ = 0.977) on the HAuCl_4_ concentration of the solution which is important for interpretation of another measurements.

### 3.3. Thermogravimetric Analysis Measurements

**Figure 4:**
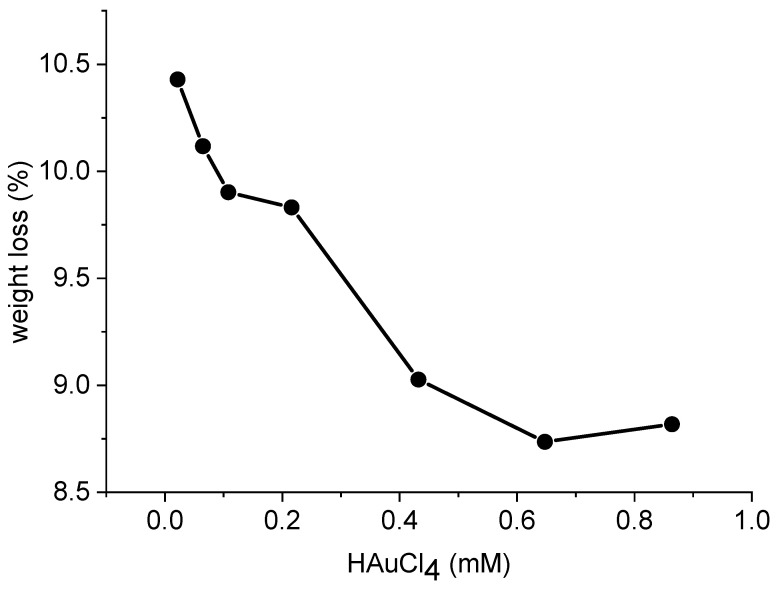
Dependence of weight loss of as-prepare films obtained from solution with different HAuCl_4_ concentration.

Free water content was determined using TGA. The amount of free water may be evaluated by the decrease in the sample weight during the heating scan. The loss in weight at 120 °C was taken to be the result of water evaporation. TGA measurements in as-prepare films (Figure 4) have shown that moisture content in films is dependent on the HAuCl_4_ concentration (or on concentration of AuNPs, as shown on Figure 3). Additionally, TGA measurements were performed on films annealed for 30 min in the measurement system at 120 °C, with subsequent cooling to room temperature in the cell. Thereafter, the second scan was performed. In such annealed films, the water content was about 0.2 wt%.

### 3.4. Dynamic Mechanical Analysis

DMA technique was used to analyze the kinetic properties of the material by measuring deformation/tension that is generated in the sample with respect with time.

As in TGA, DMA measurements were performed under the same heat treatment conditions on as-prepared samples. Additionally, DMA measurements were carried out on annealed samples at 120 °C during 30 min. with subsequent cooling to room temperature in the measurement cell. Thereafter, the second scan was performed. In such annealed films, the water content was about 0.2 wt% according to TGA measurements.

**Figure 5:**
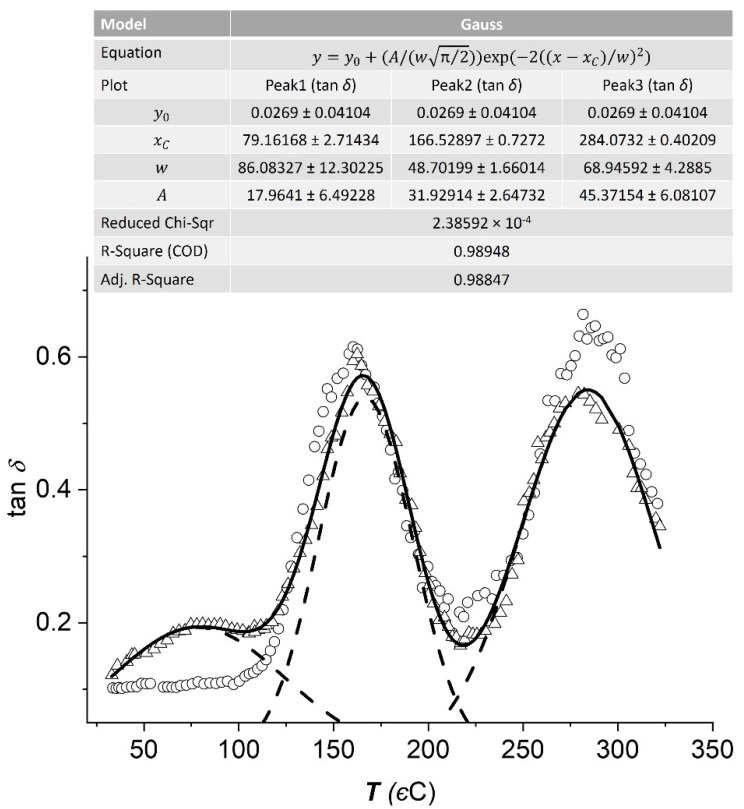
tan δ versus temperature for CS/AuNPs film obtained from solution with 0.2 mM of HAuCl4 during first scan with moisture content about 10.4% (open triangular) and second scan with moisture content less 0.2% (open circles). Continuous line demonstrates results of fitting, dash lines—position of peaks, obtained from fitting (R^^2^ = 0.988).

Figure 5 shows tan δ versus temperature for the CS/AuNP film obtained from the solution with 0.2 mM of HAuCl_4_ during the first scan with a moisture content of about 10.4% (as-prepared film) and the second scan (annealed film at 120 °C with subsequent cooling to room temperature) with a moisture content of less than 0.2%. Points are experimental data, the continuous line was obtained by fitting the experimental data using a Gauss distribution, and the dashed lines are used to visualize the positions of the peaks obtained from the fitting with R^^2^ = 0.988.

**Figure 6:**
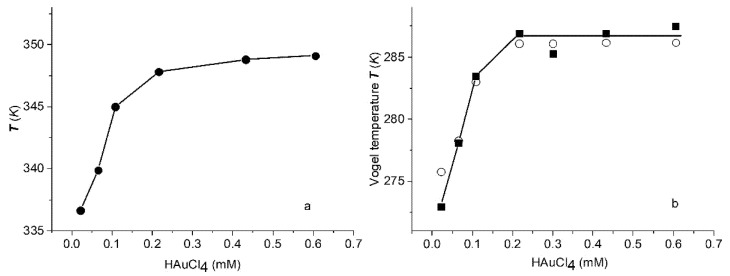
Dependencies on HAuCl4 concentration of: (**a**) the first peak position obtained from DMA measurements and (**b**) Vogel temperature obtained from conductivity (open circles) and dielectric (black squares) measurements. All measurements were carried out on as-prepare films.

All CS/AuNP as-prepared films exhibit three tan δ peaks. The first peak (at 79 °C) was observed only in as-prepare films with high water content. The position of first peak depends on the HAuCl_4_ concentration or concentration of AuNP. Figure 6a shows the position of the first peak relative to the HAuCl_4_ concentration of the solution. The positions of the second (at 166 °C) and third peaks at the temperature of 284 °C practically do not depend on the AuNP concentration. In all films with low water content (less than 0.2 wt%), the first peak disappeared (Figure 5). Note that the same two peaks’ positions at about 60–100 °C and 160 °C and their dependence on nanoparticle concentration have been observed in neat CS and CS/AuNP films [38,48]. Our previous investigation [43] showed that peak at about 79 °C related to glass transition temperature; at about 160 °C in the tanδ versus temperature dependence for neat CS is related to σ-relaxation (the nature of this relaxation will be discussed later) and peak at 284 °C appear due to degradation of chitosan. Additionally, concluding from DMA measurements about the nature of the observed relaxations processes is challenging [39,49]. Therefore, additional dielectric spectroscopy measurements were performed on the CS/AuNP films.

### 3.5. X-ray Diffraction

**Figure 7:**
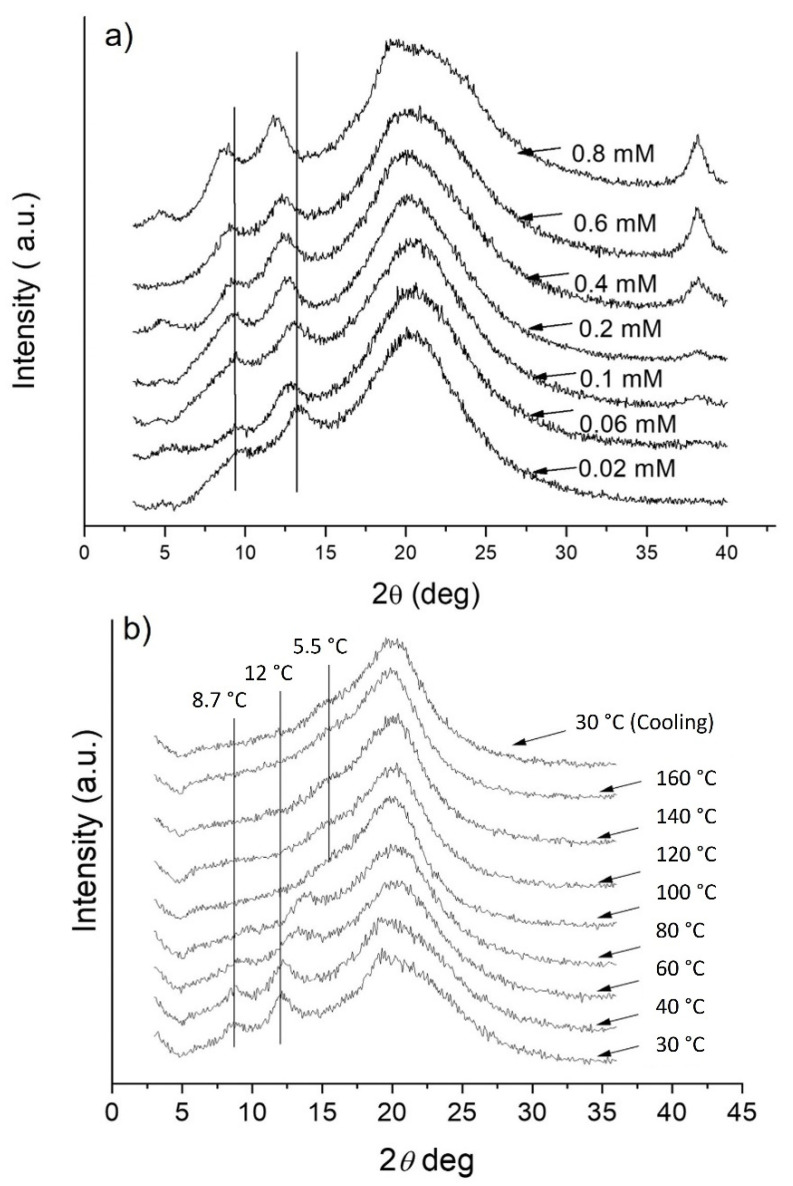
X-ray spectra: (**a**) for as-prepare films at the room temperature obtained from solution with HAuCl_4_ concentration indicated on graph and (**b**) for film obtained from solution with 0.21 mM of HAuCl_4_ at the temperature indicated on graph.

Figure 7a shows XRD spectra for the as-prepared films at room temperature obtained from solutions with different HAuCl_4_ concentrations. All spectra show diffraction lines around 2θ = 8.7°, 12° and 20° and a weakly resolved line at 21.8°. The peaks around 8.7°, 12°, and 21.8° should be assigned to the hydrated form of CS which correspondent to crystal forms II [50,51,52,53,54,55,56]. The strongest reflection that appeared at around 20° corresponding to the amorphous phase. With increasing HAuCl_4_ concentrations, the diffraction lines around 8.7° and 12° shift to lower 2θ values. Additionally, at high HAuCl_4_ concentrations, a reflection appeared at 2θ = 38.18°, corresponding to the reflection from Au nanoparticles.

Figure 7b shows XRD patterns of the film obtained from the solution with 0.21-mM HAuCl_4_ at temperatures indicated on the graph. With increasing temperatures, the diffraction line corresponding to the hydrated form of CS (at 2θ = 8.6° and 12°) shifts to higher 2θ and disappears at 100 °C. The same peak disappears at high temperatures at 2θ = 21.8°. Additionally, at this film temperature, we observed a not well-resolved diffraction line around 2θ = 15.5°. This line corresponds to the dehydrated or dry form of CS or CS salt [43,44]. After cooling to room temperature (Figure 7b, upper pattern), all films were in the dehydrated form.

XRD measurements have shown that with increasing of HAuCl_4_ concentration and temperature change crystalline structure of nanocomposites due the change water content. More detail analysis of XRD measurements will be done in the Discussion.

### 3.6. Conductivity Measurements

**Figure 8:**
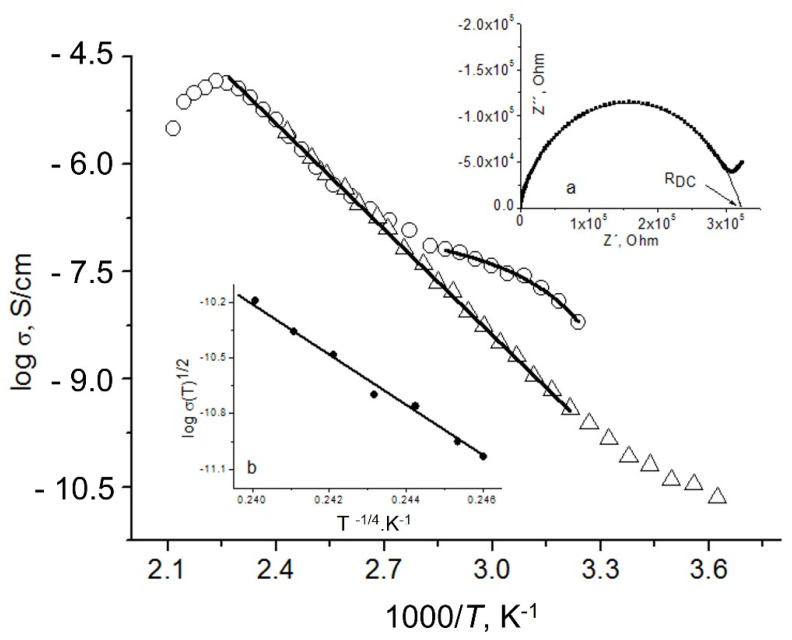
Temperature dependence of DC conductivity in as-prepare (open circles) and annealing films (open triangular) obtained from solution with 0.3 mM of HAuCl_4_. Continuous line-results of fitting. Insert (**a**) shows impedance spectra and fitting procedure using for obtained R_DC_. Insert (**b**) shows dependence log σ(T)^1/2^ vs T^−1/4^.

Figure 8 shows the temperature dependence of the DC electrical conductivity for the as-prepared film obtained from the solution with 0.3-mM HAuCl4 (water content 9.5%) and annealed into the vacuum cell before measurements at 120 °C. DC conductivity (σ_DC_) was calculated from the equation *σ_DC_* = *d*/(*R_DC_ × S*), where d and S are the thickness and area of the sample, respectively. R_DC_ was obtained by fitting a high-frequency semicircle of the impedance spectra before interception with real parts of impedance as shown in the inset of Figure 8 [49,57].

The temperature dependencies of DC conductivity show three different behaviors in both films:

- A nonlinear behavior in the as-prepared film with water content between 8.5% and 10.4% in the temperature range of 25–70 °C. The nonlinear VFT behavior observed in the temperature conductivity measurements is a well-known feature of the α-relaxation process related to glass transition (see Equation (2)).
- An Arrhenius-type linear dependence (see Equation (3)) in the temperature range of 70–150 °C for the as-prepared film and in the temperature range 33–150 °C for the annealed film, with the same activation energy of 103.2 kJ/mol.
- A negative slope in both films at temperatures greater than 160 °C, corresponding to the beginning of degradation [38,39].
- Nonlinear dependence for the annealed film in the temperature range 0–33 °C.

In the dry films with water content less than 0.2 wt% (according to the TGA measurements), the VFT behavior vanishes, and a linear behavior takes its place. Thus, conductivity can be well described by the Arrhenius equation from 33 °C until the onset of degradation with the same slope as in the wet films (open triangular on Figure 8).

**Figure 9:**
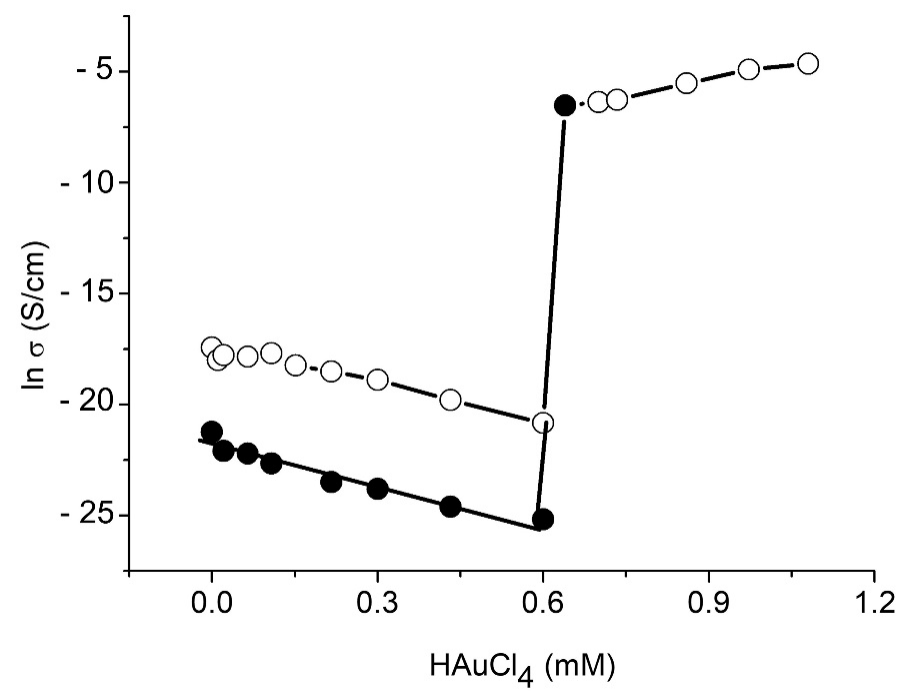
Dependence of DC conductivity of wet (open circles) and dry (black circles) films at the room temperature on the concentration of HAuCl_4_**.**

The same type of dependence of DC conductivity on reciprocal temperature is shown for all films obtained from the solution with 0–0.6 mM of HAuCl_4_. In the films obtained from solutions with higher HAuCl_4_ concentrations, conductivity practically did not depend on temperature. This is because at higher concentrations, the percolation effect was observed. Figure 9 shows the dependence of DC conductivity in the as prepared and dry films at room temperature as a function of the HAuCl_4_ concentration of the solution (or AuNPs concentration). An abrupt increase in conductivity with increasing concentrations and subsequent saturation is typically observed for the percolation phenomena and may be attributed to the formation of conductivity paths in the composite formed by nanoparticles, citrate, and NaCl (see discussion below). Such behavior of the DC conductivity in percolation systems has been reported in various types of disordered polymer–conductor composites [57]. Additionally, the conductivity of the wet films is higher than that of the dry films.

### 3.7. Dielectric Measurements

**Figure 10:**
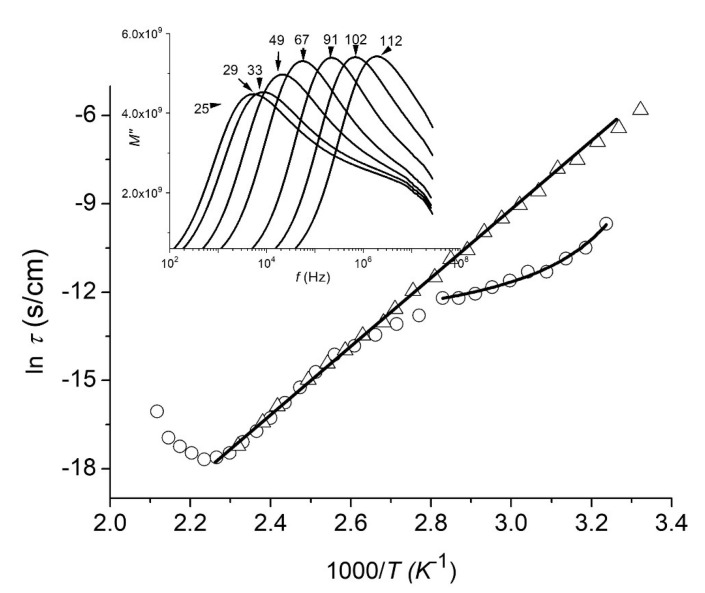
Temperature dependence of relaxation time in as-prepare (open circles) and annealing films (open triangular) obtained from solution with 0.3 mM of HAuCl4. Continuous line-results of fitting. Insert shows frequency dependence of the imaginary part of the electric modulus at the temperature indicated on graphs.

Dielectric relaxation measurements were performed and analyzed using the complex electrical modulus (see Equation (5)). The change in the imaginary parts of the electric modulus versus frequency is shown in the inset of Figure 10 for films obtained from the solution with 0.3-mM HAuCl_4_ at the temperatures indicated on the graphs. In the temperature range of 25–70 °C, the peaks have less amplitude than those of peaks in the temperature range of 70–150 °C. When the temperature increases, the peaks shift to higher frequency ranges. Figure 10 shows the dependencies of the relaxation time on the reciprocal temperature calculated from the peaks’ locations. As in the case of conductivity measurements, relaxation times show three different dependencies in films with water contents greater than 0.2 wt%:

(1) Nonlinear behaviors in the temperature range of 22–70 °C (for peaks with lower amplitude), which is well described by the VFT relationship (see Equation (2) with τ, $\tau_{0}$ instead of σ, $\sigma_{0}$ respectively).
(2) Arrhenius-type linear dependencies in the temperature range of 70–150 °C for the as-prepared film and in the temperature range of 22–150 °C for the annealed film, with an activation energy of 102.4 kJ/mol (see Equation (3) with τ, $\tau_{0}$ instead of σ, $\sigma_{0}$ respectively).
(3) At temperatures greater than 160 °C, a negative slope has been observed, corresponding to the beginning of degradation [39,49].

As in the conductivity measurements in the dry films, the VFT behavior vanishes (open triangular on Figure 10), and a linear behavior takes its place, with the same slope as in the wet film. As in the conductivity measurements, such dependence of relaxation time on the reciprocal temperature is shown for all films obtained from the solution with 0–0.6 mM of HAuCl_4_. In the films obtained from solutions with higher HAuCl_4_ concentrations, relaxation cannot be observed due to the high conductivity contribution.

Note that the Vogel temperature in the wet films and the activation energy in the dry films obtained from conductivity and dielectric relaxation measurements are approximately the same (Figure 6b) and correlate well with those from the DMA measurements. Additionally, the values of the Vogel temperature and the activation energy, as in the position of the first peak in the DMA measurements, are dependent on the HAuCl_4_ concentration of the solution (Figure 6a).

## 4. Discussion

The synthesis of Au nanoparticles in CS using SC is complex because, in this case, HAuCl_4_, CS, and SC are ionized simultaneously. Therefore, during the reaction, several electrostatic interactions between CS, SC, and the Au salt can be observed. A previous study [58] proposed that the nanoparticles obtained during synthesis are surrounded by sodium succinate, which provides stability to the structure and reduces the agglomeration effects. Additionally, according to Refs. [59,60], sodium succinate forms hydrogen bonds with the polymer matrix. Based upon these results, we can propose the following reaction between CS, HAuCl_4_, SC, and acetic acid:

Because the nanocomposites in this work have been prepared with the stoichiometric ratio of 1 mol of HAuCl_4_ for 2 mol of SC (not 4 mol of SC as necessary to achieve a full reaction between HAuCl_4_ and SC [32]), some HAuCl_4_ salt will be in the films.

Additionally, in hydrated, as-prepared CS films, conductivity sufficiently increases [49,61]. This effect was explained by the protonation of free amino groups and the formation of some hydroxide ions, which give a contribution in the ionic current according to the following reaction:

(6) $${\mathrm{NH}}_{2}+H_{2}O\;↔{\mathrm{NH}}_{3}+{\mathrm{NH}}^{-}$$

The appearance of additional OH^−^ ions in the as-prepared films explains why its DC conductivity is greater than the conductivity of the dry annealed films (Figure 9).

Additionally, according to Ref. [62] conductivity of CS demonstrates Grotthuss mechanism: protons originating from the protonated amino groups can move along the hydrated molecule via hydrogen-bond network hopping process through the breaking/formation of hydrogen bonds. According to the reaction between CS, HAuCl_4_, and SC proposed earlier, the number of NH_3_^+^ ions decrease due to the formation of bonds with AuNPs and sodium succinate. With increasing the concentrations of AuNP decreases the number of free NH_3_^+^ ions and subsequently proton conductivity which is responsible for the decreasing DC conductivity observed in Figure 10 at the films with low concentration of AuNP. This conclusion is confirmed in FTIR measurements by the shift to higher wavenumbers and the deformation of bonds corresponding to NH_3_^+^ groups due to the interaction between amine groups and metal particles [47,48].

**Scheme 1:**
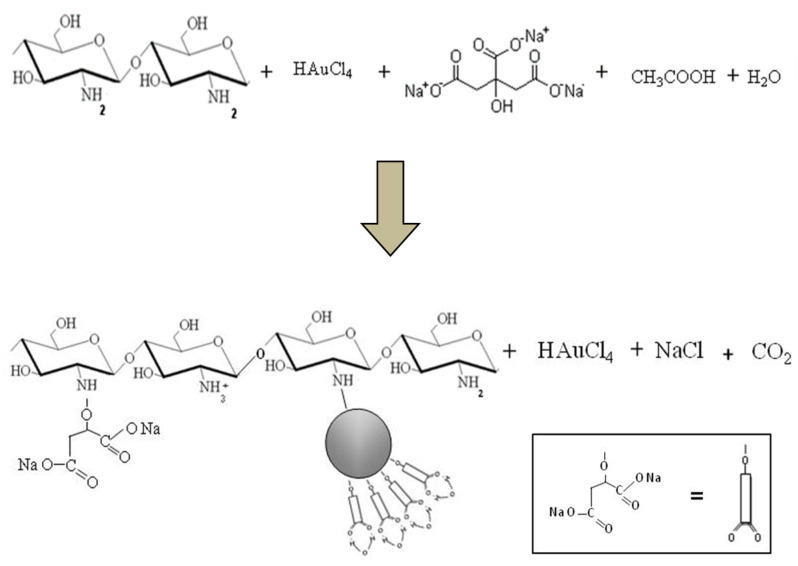
Proposed reaction between CS, HAuCl_4_, SC, and acetic acid.

At a HAuCl_4_ concentration of about 0.6 mM, the conductivity of both the wet and dry films sharply increased by six orders (from 10^−9^ to 10^−3^ S/cm), corresponding to the percolation effect. However, according to our micrograph (Figure 2b), no physical connection between Au nanoparticles is observed. According to the reaction proposed above (Scheme 1), salts of HAuCl_4_ and NaCl exist in films. Thus, we can propose that the percolation effect can be related to the appearance of a conductivity pathway between AuNPs and salts.

The next important issue in this investigation is the nature of the relaxation processes observed in the CS/AuNP composites. The nonlinear VFT behavior observed from the plots of conductivity and relaxation time versus 1000/T (Figure 8 and Figure 10) is a well-known feature of the α-relaxation process related to glass transition. This relaxation process is highly dependent on moisture content, as in these figures. In the case of dry films (annealed before measurements at 120 °C, with moisture content about 0.2%) in DMA, conductivity, and dielectric spectroscopy measurements, the α-relaxation process vanishes, and glass transition is not detected. Thus, the first peak in the DMA measurements corresponds to the glass transition temperature. Such effect has also been observed in pure CS films [40]. The obtained results suggest a plasticizing effect of water on composites. This conclusion is confirmed by XRD measurements. The as-prepared films belong to the hydrated form of CS. The annealed films correspond to the dehydrated or dry form of CS or CS salt. Therefore, the glass transition phenomena were observed only in the hydrated composites.

Additionally, the α-relaxation process in the CS/AuNP films depends on the solution from which the films were obtained. The Vogel temperature is the apparent activation temperature of the α-relaxation in many polymers; T_0_ is usually 50–70 K lower than glass transition temperature [40,62,63]. Comparing the glass transition temperature T_g_ obtained from the DMA measurements (Figure 6a) with the Vogel temperature T_0_ obtained from the conductivity and dielectric spectroscopy measurements (Figure 6b), the following relaxation for the CS/AuNP films can be obtained: T_g_ = T_0_ + 61.9 ± 0.7 K.

Note that the dependencies of the glass transition temperature (DMA measurements, Figure 6a) and the Vogel temperature (Figure 6b) demonstrated inverse properties compared with the dependence of water content in the films (Figure 4). The decreasing moisture content in the films (with increasing HAuCl_4_ and SC concentrations of the solution) is responsible for the increasing glass transition temperature (Figure 6). According to Refs. [26,39], CS has functional groups such as amines and amides, which can bond with water molecules. However, the protonated amino groups of CS and the carboxylate groups of SC form ionic bonds [63]. We proposed that the same interaction can be observed in the CS–sodium succinate system, according to reaction (3). At the same time, the Au nanoparticles are attached by electrostatic forces to the NH_3_^+^ group [32]. Thus, with increasing HAuCl_4_ and SC concentrations, the number of free NH_3_^+^ groups decrease, which is responsible for bonding with water molecules. Additionally, the increasing Au concentration in films is responsible for the increasing interplanar distance and shift of the diffraction lines around 8.7° and 12° to lower 2θ values in the hydrated form of the composites.

In the case of dry films (with a water content of about 0.2 wt%) below the percolation threshold in all temperature ranges below degradation and in wet functionalized films between 70 °C and 160 °C, in both the conductivity and relaxation time plots, an Arrhenius-type behavior (Figure 8 and Figure 10) was observed with activation energies between 80 and 118 kJ/mol. These values agree with previous reports for CS and other polysaccharides [26,52]. This relaxation process is called the σ-relaxation and can relate to the local diffusion process of ions between high potential barriers in disordered systems [64]. So, the second peak in the DMA measurements (Figure 5) corresponds to σ-relaxation. Moreover, the activation energies obtained from conductivity and dielectric spectroscopy measurements are approximately the same.

Additionally, in all dry films below the percolation threshold in the temperature range of 0–33 °C in the dependence of conductivity on the temperature (Figure 9), nonlinear dependence was observed. The applicability of the VRH model (see Equation (4)) is examined by plotting the experimental results in the form of ln*σ*(T)^1/2^ versus T^−γ^ [41,42]. If proposed that CS/AuNPs demonstrate the three-dimensional of the transport process dependence of ln*σ(T)*^1/2^ versus T^−1/4^ must be linear that demonstrate experimental (inset b in Figure 8).

## 5. Conclusions

CS/AuNPs thin films have been synthesized through the chemical reduction of HAuCl4 in SC and CS solutions. The dielectric and dynamic mechanical behaviors of CS/AuNP films were investigated as a function of moisture and HAuCl_4_ content. We showed two relaxation processes in the nanocomposites. First, a primary α-relaxation process related to glass transition is observed in the wet CS/AuNP films. It shifts to higher temperatures with decreasing moisture content; however, in dry composites (about 0.2-wt% moisture content), glass transition cannot be observed. This primary α-relaxation process is also affected by the HAuCl_4_ contents of the solution from which the films were obtained because of the interaction between CS, sodium succinate, and Au nanoparticles, which is responsible for changes in the water content in the CS/AuNP films.

A second relaxation process was observed from 70 °C to the onset of thermal degradation (160 °C) in the wet films and from 33 °C to the onset of degradation in the dry films. This relaxation is identified as the σ-relaxation and can be related to the local diffusion process of ions between high potential barriers in disordered systems. σ-relaxation exhibits a normal Arrhenius-type temperature dependence with activation energies between 80 and 118 kJ/mol which is typically for all polysaccharides.

In dry films below the percolation threshold in the temperature range of 0–33 °C in the dependence of conductivity on temperature, nonlinear dependence was observed, which was explained using the VRH model.

At a HAuCl_4_ concentration of about 0.6 mM, the conductivity of both the wet and dry films sharply increased by six orders (from 10^−9^ to 10^−3^ S/cm), corresponding to the percolation effect, which can be related to the appearance of a conductivity pathway between the AuNPs, HAuCl_4_, and NaCl.

In conclusion, in this work we report a systematic study of such composites and propose an in-depth interpretation of the mechanisms involved in the observed behaviors. More importantly, an in-depth analysis of the mechanisms affecting the variation in conductivity as a function of the HAuCl_4_ concentration and water content is presented. We have shown that dependent on the HAuCl_4_ concentration and water content can be obtained nanocomposites with different conductivity which can affected on properties and sensibility of sensors. This conclusion has been confirming our previously investigation of sensibility of CS/AuNP sensor for detection of Cu ions in water: the best sensibility has been obtained in films with concentration of nanoparticles near percolation threshold [65,66].

## Data Availability

Not applicable.
